# RANTES mediates kidney ischemia reperfusion injury through a possible role of HIF-1α and LncRNA PRINS

**DOI:** 10.1038/srep18424

**Published:** 2016-01-04

**Authors:** Tung-Min Yu, Kalaiselvi Palanisamy, Kuo-Ting Sun, Yuan-Ji Day, Kuo-Hsiung Shu, I-Kuan Wang, Woei-Cherng Shyu, Ping Chen, Yuh-Lien Chen, Chi-Yuan Li

**Affiliations:** 1 Graduate Institute of Clinical Medical Science, China Medical University, Taiwan; 2 Division of Nephrology, Department of Internal Medicine, Taichung Veterans General Hospital, Taiwan; 3 Department of Pediatric Dentistry, China Medical University Hospital, Taiwan; 4 School of Dentistry, China Medical University, Taiwan; 5 Department of Anesthesiology, Chang Gung Memorial Hospital, Taiwan; 6 Graduate Institute of Clinical Medical Sciences, Chang Gung University, Taiwan; 7 Division of Nephrology, China Medical University Hospital, Taiwan; 8 Graduate Institute of Immunology, China Medical University, Taichung, Taiwan; 9 Center for Neuropsychiatry, Department of Neurology, China Medical University Hospital, Taichung, Taiwan; 10 Thalassemia Research Institute, The First Affiliated Hospital, Guangxi Medical University, China; 11 Department of Anatomy and Cell Biology, National Taiwan University, Taipei, Taiwan; 12 Department of Anesthesiology, China Medical University Hospital, Taiwan

## Abstract

RANTES (Regulated on activation, normal T-cell expressed and secreted), recruits circulating leukocytes and augments inflammatory responses in many clinical conditions. Inflammatory responses in ischemia-reperfusion injury (IRI) significantly affect the unfavorable outcomes of acute kidney injury (AKI), and that infiltrating immune cells are important mediators of AKI. However, the significance of RANTES in AKI and whether hypoxia-induced LncRNAs are involved in the regulatory process of AKI are not known. Here we show that, in the kidney IRI mice model, significant RANTES expression was observed in renal tubular cells of wild type mice. RANTES deficient (RANTES^−/−^) mice showed better renal function by reducing the acute tubular necrosis, serum creatinine levels, infiltration of inflammatory cells and cytokine expressions compared to wild type. *In vitro,* we found that RANTES expression was regulated by NF-κB. Further, renal tubular cells showed deregulated LncRNA expression under hypoxia. Among HIF-1α dependent LncRNAs, PRINS (Psoriasis susceptibility-related RNA Gene Induced by Stress) was significantly up regulated in hypoxic conditions and had specific interaction with RANTES as confirmed through reporter assay. These observations show first evidence for RANTES produced by renal tubular cells act as a key chemokine in AKI and HIF-1α regulated LncRNA-PRINS might be involved in RANTES production.

Infiltration of leukocytes into ischemic tissues in kidney ischemia-reperfusion injury (IRI) has been associated with adverse outcomes of acute kidney injury (AKI)^1^,^2^. Following ischemic injury, rapid influx of circulating immune cells accompany with the release of several inflammatory molecules such as cytokines, chemokines and complement^3^. Chemokines and their receptors play a crucial role in the inflammatory responses which greatly enhances the recruitment of immune cells including neutrophils, monocytes and lymphocytes^4^,^5^. Several chemokines have been demonstrated to be involved in kidney inflammatory response in IRI. For example, IL-8/CXCL8, MIP-2/Gro-β/CXCL2 and Gro-α/CXCL1 are suggested to be associated with marked neutrophil infiltration after IRI, and the CCL subfamily (MCP-1/CCL2) and CX3CL subfamily (fractalkine/CX3CL1) may have specific effects on monocytes and monocyte-derived lineages^5^. Therefore, chemokines has been suggested to play an important role in affecting the inflammatory reaction in IRI.

RANTES (Regulated on Activation Normal T cell Expressed and Secreted, also known as CCL5), a member of the CC-chemokine family, is well-known for its potent chemo-attractant effect on immune cells. It could be secreted by various tissues and has been shown to simultaneously orchestrate the recruitment of several immune cells, such as neutrophils, monocytes and lymphocytes^4^,^5^,^6^. An extensive level of RANTES on cell surfaces during IRI has shown to act as a powerful activator of leukocytes^7^. In many clinical situations, including atherosclerosis, stroke and myocardial IRI, RANTES has been demonstrated to play a dominant role in infiltrating inflammatory cells and concomitantly promotes the release of other inflammatory cytokines in the site of post-ischemia inflammatory injury^7^,^8^,^9^,^10^,^11^. RANTES participates in immune responses in myocardial infarction by recruiting neutrophils and monocytes thereby mediate cerebral ischemia^7^,^10^. However, data regarding the role of RANTES in ischemia kidney injury is not very well known.

Emerging evidence show long non coding RNAs (LncRNA’s) as key regulatory molecules in cellular signaling^12^. These RNAs contain nucleotides with size greater than 200, and functions in epigenetic regulation, transcriptional, post-transcriptional and translation events^13^. Aberrant expression of LncRNA’s has been implicated under various diseases^12^. However, important LncRNA involved in regulatory process of acute kidney injury has not been explored till date.

Given its patho physiological role in many diseases caused by ischemic reperfusion and its great capacity to recruit inflammatory cells, RANTES might be relevant to the inflammatory response in acute kidney injury following IRI. The aim of this study was to 1. Investigate the role of RANTES in post-ischemia inflammatory responses and its associated renal function changes in AKI. 2. To identify functional contribution of hypoxia and LncRNA in RANTES expression.

## Results

### RANTES expression increased in kidney IRI of wild type mice

**Figure 1:**
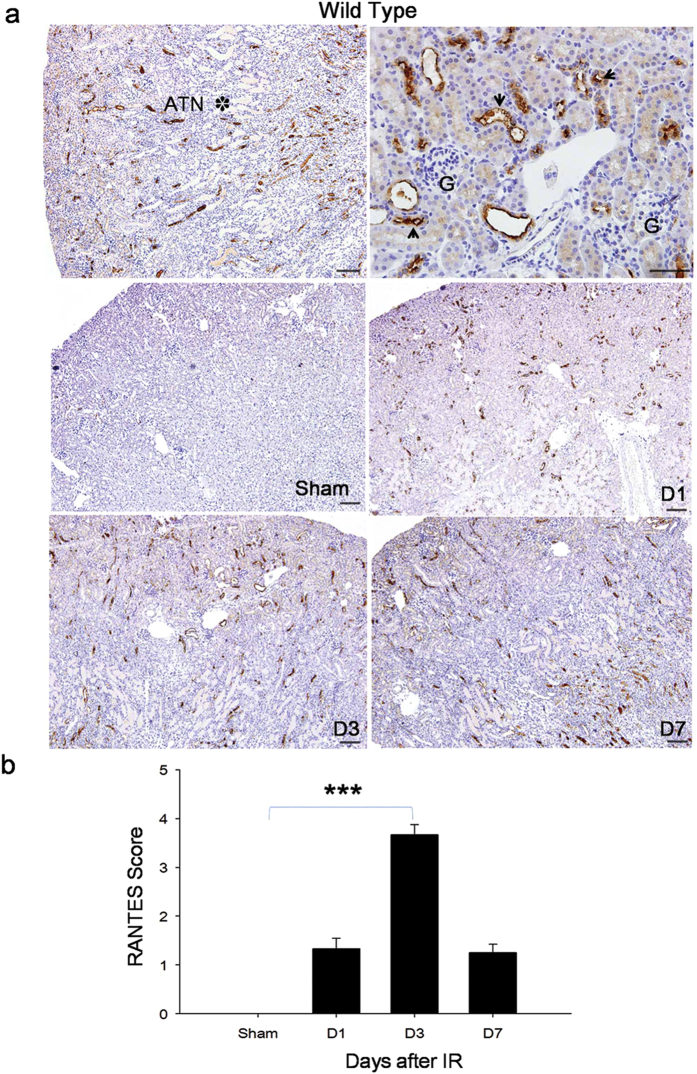
Wild type mice with kidney IRI shows increased RANTES expression. (**a**) Representative kidney sections showing increase in RANTES expression in cortical renal tubular epithelial cell (brown color as pointed by black arrow; G: glomerulus) with nearby significant acute tubular necrosis (ATN*) in wild type mice with kidney IRI as determined by immunohistochemisty staining (original magnification, x200, in left panel; x400, in right panel; scale bar - 50 μm). RANTES expression was examined on days 1, 3, 7 in wild type mice with kidney IRI by immunohistochemisty (stained as brown color, magnification, x100; scale bar - 50 μm). Throughout the course of the study a significant up regulation of RANTES expression was observed compared to sham group. (**b**) Semi quantitative analysis showed significant increase (*P* < 0.001) in RANTES expression on day 3 in kidney IRI compared to sham group. The data shown are mean ± SD; n = 6.

To determine RANTES expression in kidney IRI, we performed immunohistochemical staining on days 1, 3, and 7 in wild type mice after kidney IRI. Sham operated controls did not show any RANTES expression. However, there was significant increase in RANTES expression in renal tubular epithelial cells on day 3 (*P* < 0.001) compared to sham mice (Fig. 1).

### Renal function was better in RANTES^(−/−)^ mice with IRI

**Figure 2:**
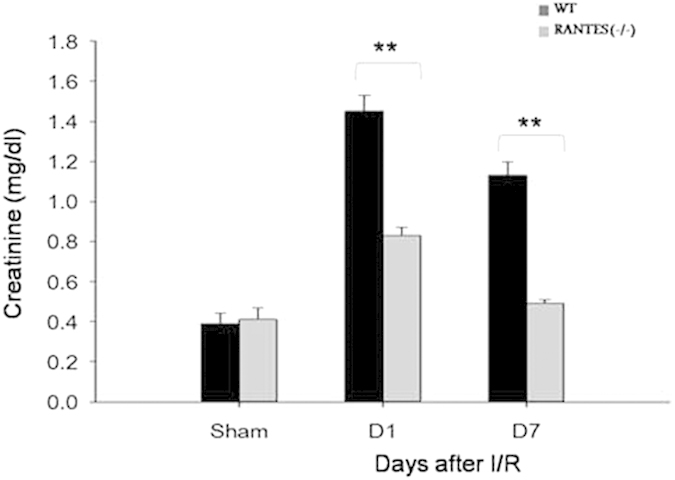
Renal function was improved in RANTES^(−/−)^ mice. Renal function was monitored in wild type and RANTES^(−/−)^ mice by determining serum creatinine on days 1 and 7 after IRI injury. RANTES^(−/−)^ mice showed significantly lower (*P* < 0.01) creatinine on days 1 and 7 after reperfusion compared with wild type mice. Sham-operated mice had normal serum creatinine. The data represented are mean ± SD. (n = 6 for days 1 and 7 group; n = 3 for sham group).

To determine the renal function, serum creatinine was measured in wild type and RANTES^(−/−)^ mice on days 1 and 7 post kidney IRI. Compared to wild mice, serum creatinine were significantly lower in the RANTES^(−/−)^ mice on days 1 and 7 (*P* < 0.01) (Fig. 2).

### Acute tubular necrosis decreased in the RANTES^(−/−)^ mice with kidney IRI

**Figure 3:**
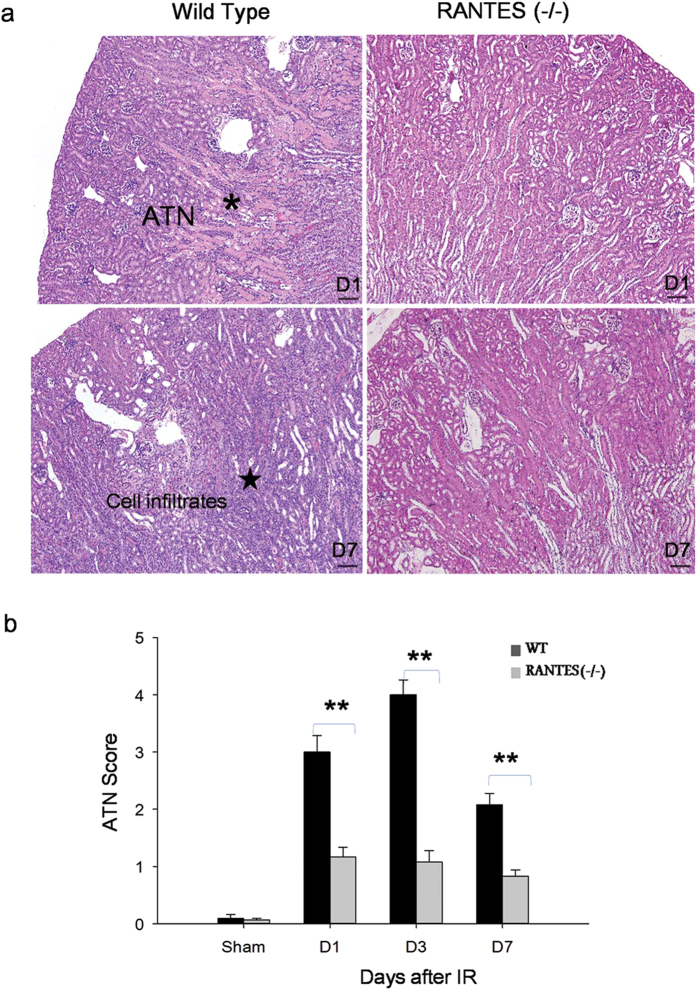
Tubular injury was significantly less in RANTES^(−/−)^ mice. (**a**) Representative sections of cortico medulla tissue from wild type mice and RANTES^(−/−)^ mice on days 1 and 7 after reperfusion (hematoxylin- and eosin-stained; magnification, x100; scale bar - 50 μm). Compared with WT mice, ATN* changes and interstitial inflammation with infiltrates was significantly attenuated in RANTES^(−/−)^ mice. (**b**) Semi quantitative analysis of renal tubular damage shows significant decrease (*P* < 0.01) of ATN score in RANTES^(−/−)^ mice compared with wild type mice in kidney IRI on days 1, 3 and 7 after reperfusion. The data represented are mean ± SD. (n = 3 to 6 per group).

To compare the tubular injury in RANTES^(−/−)^ and wild-type mice with IRI, we examined the acute tubular necrosis (ATN) score by histopathology. In the wild-type mice, extensive tubular necrosis, loss of brush border, cast formation, tubular dilation and inflammatory infiltrates was noted at the cortico medullary junction after IRI. However, tubular damage was attenuated in the RANTES^(−/−)^ mice and maintained normal kidney morphology (Fig. 3a). The results from ATN score demonstrate that the RANTES^(−/−)^ mice had lower ATN score after IRI which was statistically significant (*P* < 0.01) when compared to wild-type mice (Fig. 3b). Sham operated mice had no tubular injuries (Data not shown).

### Impaired infiltration of inflammatory cells after IRI in RANTES^(−/−)^ mice

**Figure 4:**
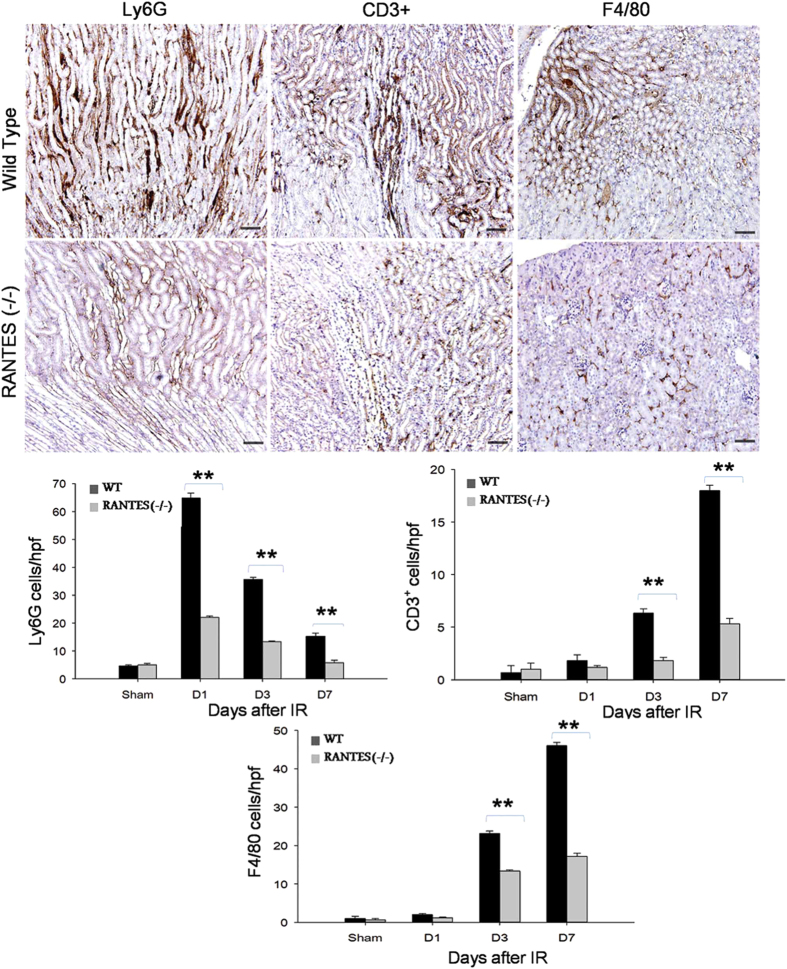
Accumulation of interstitial inflammatory cells was reduced in RANTES^(−/−)^ mice. Representative sections of kidney tissues by immunohistochemisty stained for neutrophils (Ly6Gcells/ hpfs), T lymphocytes (CD3^+^ cells/ hpfs) and macrophages (F4/80/ hpfs) on day 7 after reperfusion (stained as brown color, magnification, x 200; scale bar - 50 μm). Inflammatory infiltrates was significantly reduced (*P* < 0.01) in the interstitium of cortical-medullary zones of RANTES^(−/−)^ mice compared to wild type mice with kidney IRI.

To investigate recruitment of inflammatory cell infiltrates in RANTES^(−/−)^ mice, we performed immunohistochemisty to detect neutrophils, lymphocytes and macrophages on days 1, 3 and 7 after kidney IRI. A substantial number of interstitial cells accumulated in the cortical-medullary area of the wild-type mice in IRI. In addition, a significantly less infiltrating cells including Ly6G, CD3^+^, F4/80 (P < 0.01) were noted in the RANTES^(−/−)^ mice compared with wild-type mice (Fig. 4).

### Proinflammatory cytokines and chemokines production were attenuated in RANTES^(−/−)^ mice with kidney IRI

**Figure 5:**
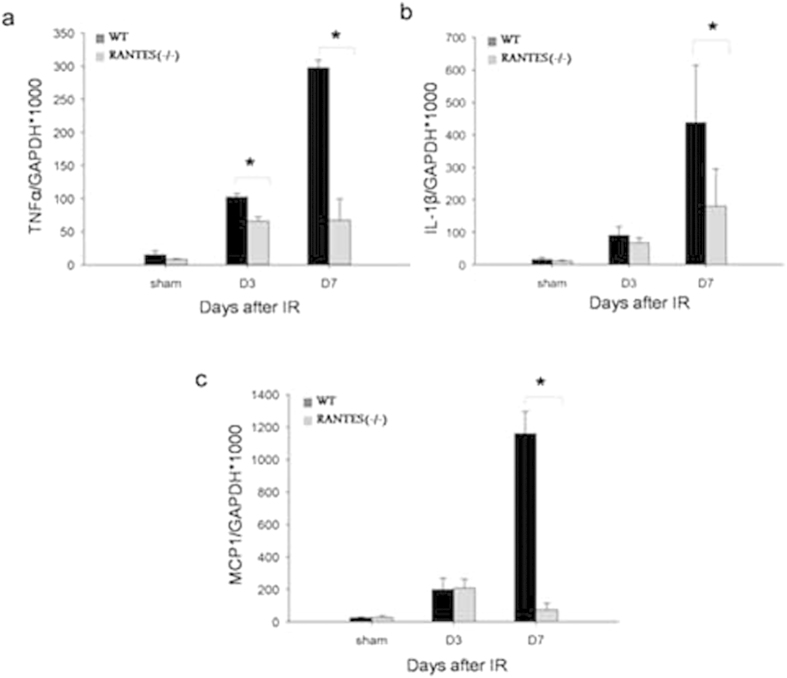
Proinflammatory cytokines and chemokines were down regulated in RANTES^(−/−)^ mice. mRNA expression of pro-inflammatory cytokines and chemokines were measured on days 3 and 7 after reperfusion by quantitative real-time PCR. mRNA expression of TNF-α (**a**), IL-1β (**b**) and MCP-1 (**c**) after kidney IRI was significantly reduced (*P* < 0.05) in RANTES^(−/−)^ mice compared with wild type mice. The results have been normalized by expressing the number of transcript copies as a ratio to GAPDH (n = 3 to 6 per group).

To determine the inflammatory cytokines after IRI, we measured the mRNA expression of TNF-α, IL-1β and MCP-1 in the kidneys. An increase in TNF-α, IL-1β and MCP-1 on days 13 and 7 after IRI was found in wild mice compared to sham operated mice. However, a significant decrease (*P* < 0.05) in inflammatory cytokines expression were noted in the RANTES^(−/−)^ mice compared to wild type mice (Fig. 5a–c).

### NF-κB-p50 regulates RANTES expression

**Figure 6:**
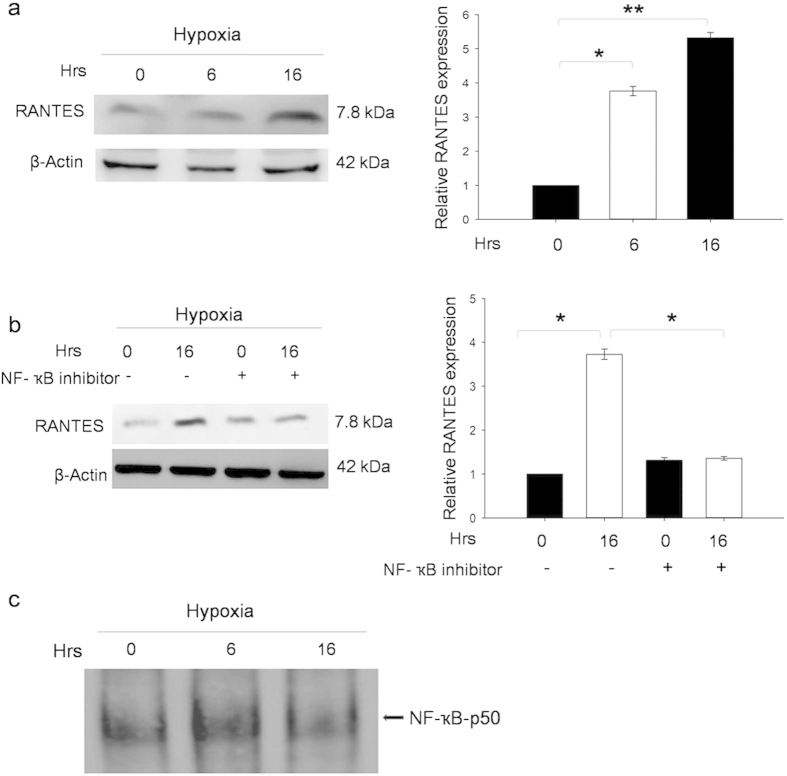
NF-κB mediates RANTES expression under hypoxic condition. (**a**) RANTES expression was induced under hypoxia. A significant increase in RANTES expression was observed at 6 hours (*P* < 0.05) and 16 hours (*P* < 0.01) compared to control under hypoxic stress. (**b**) NF-κB inhibitor down regulates RANTES expression. Cells pre-treated with NF-κB inhibitor, showed significant down regulation (*P* < 0.05) in RANTES expression. (**c**) NF-κB-DNA binding activity was increased under hypoxia stimulation at 6 hours compared with control. The assay is representative of three experiments performed in triplicates.

In order to determine hypoxia-induced RANTES expression, cultured renal tubular cells were exposed to hypoxic stress for 6 and 16 hours. Western blot results showed significant up regulation in CCL5/RANTES expression at 6 (*P* < 0.05) and 16 hours (*P* < 0.01) compared to control (Fig. 6a). Figure 6b shows pre-treatment with NF-κB inhibitor followed by hypoxic stress, significantly down regulated RANTES expression at 6 and 16 hours (*P* < 0.05) compared to control. Figure 6c demonstrates NF-κB activation under hypoxic condition through EMSA. There was increase in NF-κB-DNA binding activity in renal tubular cells under hypoxia for 6 hours.

### HIF-1α regulates LncRNA expression in hypoxic renal tubular cells

**Figure 7:**
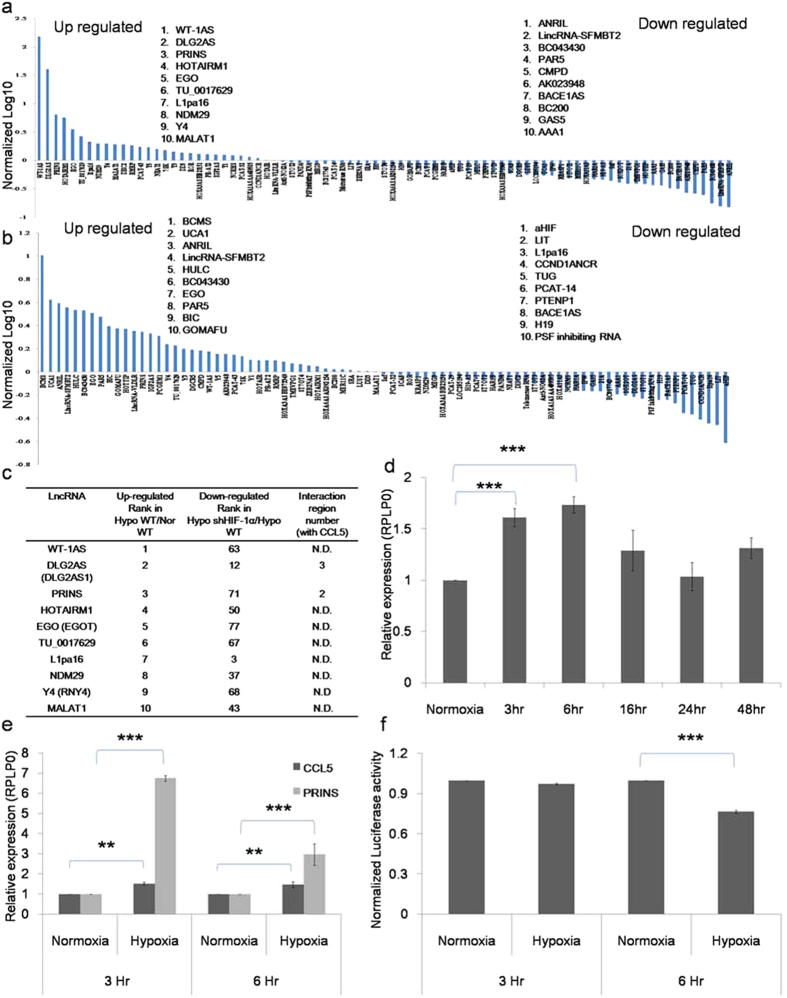
LncRNA-PRINS and RANTES interacts under hypoxia. (**a**) Deregulated LncRNA in hypoxia/normoxia condition for 24 hours. (**b**) Deregulated LncRNA in HK-2 sh HIF-1α and HK- 2 cells under hypoxia 24 hours. Briefly, cells were exposed to normoxia/hypoxia for 24 hours, after treatment RNA was isolated and Disease Related LncRNA array was performed. Each bar represents expression of Lnc-RNA relative to internal control. The results show 3 independent experiments performed in triplicates (**c**) Differentially expressed LncRNA in HIF-1α dependent manner. (**d**) Time course study on RANTES mRNA expression by Q-PCR. (**e**) Q-PCR analysis of RANTES and LncRNA-PRINS under normoxia and hypoxia 3 and 6 hour. Data are expressed as relative levels to RPLP0. The data are shown as the mean ± SD of three independent experiments performed in triplicates. (**f**) Luciferase reporter assay: The LncRNA-PRINS luciferase construct vector was transfected into WT-HK-2 cells for 24 hour and incubated under hypoxia and normoxia conditions for 3 and 6 hour respectively. Results were expressed in luciferase activity normalized to Renilla. The data are shown as the mean ± SD of three independent experiments performed in triplicates.

In order to identify whether LncRNAs are involved in AKI, we first determined hypoxia responsive LncRNA expression in HK-2- wild type and sh-HIF-1α knockdown cells using Q-PCR based Disease Related Lnc-RNA array (Systems Biosciences). Figure 7a shows deregulated LncRNAs in wild type HK-2 cells under 24 hours hypoxia/normoxia. The results reveal that hypoxia induced differential expression of LncRNAs; 28 LncRNAs up regulated (>1 fold) and 55 LncRNAs down regulated ( < 1 fold). Among the up regulated Lnc-RNAs, 7 were increased with >2 fold, WT-1AS (~150), DLG2AS1 (~40), PRINS (~6), HOTAIRMI (~5), EGO (~3), TU 0017629 and I1pa16 (~2). Figure 7b shows Lnc-RNA expression in HK-2- sh HIF-1α cells/wild type under hypoxia. Among the deregulated LncRNAs, 41 were up regulated (>1 fold) and 42 were down regulated ( < 1 fold). For determining HIF-1α dependent LncRNAs, the up regulated LncRNAs under hypoxia were analyzed for their regulation in sh HIF-1α cells. Figure 7c, shows hypoxia responsive LncRNAs and their down regulated position in HIF-1α knockdown cells.

We next attempted to identify whether HIF-1α dependent LncRNAs are involved in RANTES regulation, for this their binding potential with RANTES was determined through bioinformatics analysis^14^. The results show that among the LncRNAs, DLG2AS1 and PRINS had sequence homology with RANTES (Fig. 7c). These results highlight that DLG2AS1 and PRINS as HIF-1α regulated LncRNA which might interact with RANTES under hypoxia.

### LncRNA PRINS (Psoriasis susceptibility-related RNA Gene Induced by Stress) interacts with RANTES under hypoxia

To investigate whether DLG2AS1 and PRINS have any functional role in RANTES regulation, the mRNA expression of RANTES and LncRNA (DLG2AS1 and PRINS) transcripts were analyzed through Q-PCR analysis. Time course study (3, 6, 16, 24, and 48 hr) showed significant increase (*P* < 0.001) in RANTES mRNA expression at 3 hr and 6 hr hypoxia compared to normoxia (Fig. 7d). Further, expression of DLG2AS1 and PRINS transcripts were determined at this time point. A significant up regulation of PRINS (*P* < 0.001) and RANTES expression (*P* < 0.01) appeared under hypoxic condition compared to normoxia (Fig. 7e). However, LncRNA- DLG2AS expression was not significantly regulated (Data not shown). Further, to validate interaction between LncRNA-PRINS and RANTES reporter assay was performed. For this we constructed the specific RANTES sequence having LncRNA-PRINS interacting site in pmir-GLO dual luciferase reporter at the 3′ end of firefly luciferase coding sequence. Reporter assay showed significant repression (*P* < 0.001) of luciferase activity at 6 hr hypoxia (Fig. 7f). Together these observations demonstrate that both LncRNA-PRINS and RANTES are significantly up regulated and interact under hypoxic condition.

## Discussion

Ischemic reperfusion injury aggravates acute kidney injury (AKI)^15^,^16^,^17^,^18^ and several inflammatory mediators are known to involve in AKI^5^,^19^,^20^,^21^,^22^. RANTES is one of the inflammatory mediators involved in IRI-related diseases through its potent chemo-attractant effect, and for its great capacity to recruit immune cells which results in significant inflammatory injury. However, the role of RANTES in acute kidney injury remains elusive. In the present study, we found that RANTES obviously increased in renal tubular cells which further aggravated kidney injury through recruiting inflammatory cells and leading to loss of renal function after IRI in wild type mice. Genetically deficient RANTES mice (RANTES^(−/−)^) showed better renal function by significant down regulation of RANTES expression with impairment in recruitment of inflammatory cells and production of cytokines and chemokines post kidney IRI. In addition, we found that HIF-1α as a key LncRNA regulator in renal tubular cells and show that HIF-1α responsive LncRNA-PRINS might regulate RANTES production and involve in AKI process.

The influence of RANTES has been demonstrated in several ischemic organ diseases. RANTES was found to contribute in cardiac dysfunction and heart failure in mice myocardial reperfusion injury model^10^. As in mice with brain infarction, a significantly decreased infarction area was observed in brain tissue of RANTES-deficient mice^7^. Studies on ischemic injury in arterial vessels show that local vascular smooth muscle cells (SMC) play an active role in RANTES production and involve in T-lymphocyte infiltration, which consequently aggravates arterial injury in IRI^9^,^23^. To evaluate the role of RANTES in kidney IRI, we performed the study in RANTES^(−/−)^ mice and compared the effects with that of wild type mice. We observed up regulation of RANTES expression in renal tubular cells of wild type mice throughout the study schedule post IRI on days 1, 3 and 7. A significant increase in RANTES expression on day 3 might be linked to late response of renal tubular cells during ischemic injury. Although the effect of RANTES has been determined in other kidney diseases, such as acute rejection, chronic glomerulonephritis or chronic kidney failure^24^, our study is the first to demonstrate the influence of RANTES in AKI. In addition, RANTES^(−/−)^ mice showed significant attenuation of AKI with less acute tubular necrosis and lower serum creatinine levels compared to wild-type mice.

RANTES contributes to IRI through enhancing various inflammatory responses by recruiting immune cells through cognate receptors CCR1 and CCR5^4^,^19^,^20^. Treatment with RANTES antagonists reduced tissue damage in IRI-related diseases such as myocardial infarction or brain infarction by significant decrease in infiltrating inflammatory cells, such as neutrophils, macrophages and lymphocytes^7^,^10^. In the current study, RANTES^(−/−)^ mice showed impaired recruitment of inflammatory cells such as neutrophils (Ly6G), lymphocytes (CD3^+^) and macrophages (F4/80) in kidney after IRI compared to wild-type mice. Our data suggests that the chemotactic effect of RANTES influences the infiltrating cells throughout the course of kidney IRI. In a recent study, Ko *et al.* found that infiltrating T cells with simultaneous over expression of CCR5 in kidney IRI was highly associated with AKI, such effect was alleviated by blocking CCR5^20^. It has been well recognized that CCR5 is the major cellular receptor for RANTES in monocytes; therefore, deficiency of RANTES in renal tubular cells significantly reduced cellular infiltration in kidney IRI. Although previous studies suggested that T lymphocytes are the principal effector cells for the RANTES effect, the present study showed interesting finding on contribution of other infiltrating inflammatory cells (neutrophils and macrophages) in RANTES mediated cellular infiltration in kidney IRI. We supposed that in complex post-ischemia inflammatory reaction absence of RANTES affected participation of classic chemo attractants, which highlights the crucial role of RANTES in kidney IRI.

In addition, RANTES has been assumed to orchestrate inflammatory cytokines production during the post-ischemia inflammatory response in IRI. RANTES^(−/−)^ mice or mice treated with anti-RANTES mAb showed significant reduction in inflammatory cytokines including IL-6, IL-10, TNF-α, MCP-I in IRI diseases, such as myocardial ischemia, stroke and hypoxia-related apnea syndrome^10^,^7^,^9^. In our study, the qPCR data revealed that pro-inflammatory cytokines including TNF-α, IL-1β and MCP-1 (CCL2) decreased significantly on days 3 and 7 in the RANTES^(−/−)^ mice. Of note, the level of MCP-1 as well as that of other cytokines significantly decreased on day 7 in the RANTES^(−/−)^ mice compared to the wild-type mice. A more profound decrease in the inflammatory responses with associated less renal injury observed in the RANTES^(−/−)^ mice than in the wild-type mice. The present study observations were consistent with previous studies using RANTES blockers^9^,^10^,^25^. Taken together, we suggest that the expression of RANTES in renal tubular epithelial cells indicates a cellular inflammatory response between tubular endothelial cells and infiltrating leukocytes through releasing multiple inflammatory mediators that results in an overwhelming immunological injury in kidney IRI.

Accumulating evidence suggests that RANTES expression is highly associated with NF-κB signaling and both of them act coordinately to influence inflammatory cell recruitment in several inflammatory diseases. Studies show that RANTES expression is dependent on NF-κB activation by their subsequent binding to the RANTES promoter^9^,^23^. RANTES production in vascular SMC critically depends on the NF-κB signaling pathway in ischemic vascular injury and hypoxic artery remodeling^9^. To explore whether the NF-κB signaling pathway is involved in RANTES production in renal tubular cells, a hypoxic *in vitro* study was performed. The study showed increased NF-κB-p50 DNA binding at 6 hours post hypoxic stress with increased CCL5/RANTES expression at 16 hours. In addition, the expression of RANTES was significantly decreased when renal tubular cells were treated with NF-κB inhibitor under hypoxia. Our findings suggest that NF-κB is associated with the production of RANTES in renal tubular cells suffering from hypoxia.

Emerging reports show that long non-coding transcripts (LncRNAs) regulate protein coding genes in human diseases^26^. To date hypoxia regulated long non-coding transcripts have been largely identified in various cancers. Takahashi *et al.* (2014) demonstrated that hypoxia responsive LncRNA-ROR promotes cell survival in HCC cells^27^. Yang *et al.* found that down regulation of LncRNA-LET has been associated in hypoxia-induced cellular invasion in hepatic cancer^28^. Little is known regarding LncRNAs in ischemic injury. Previous studies show deregulated LncRNA’s in focal ischemia and regulatory function of AK139328 in liver ischemia/reperfusion injury^29^,^30^. Recent study by Lorenzen *et al.* (2015) showed that circulating levels of LncRNA- TapSaki as a major predictor of mortality in AKI patients^31^. However, the importance of LncRNAs in relation to AKI process is unknown. Here we report firstly that, renal tubular cells differentially express LncRNA’s under hypoxic conditions and among the 83 disease related LncRNAs, 28 LncRNAs were up regulated and 55 LncRNAs were down regulated.

HIF is a major regulatory protein in hypoxia-induced pathogenesis; here we identified that PRINS as HIF-1α dependent LncRNA and PRINS was significantly up regulated throughout the hypoxic conditions. Previous reports show that, PRINS as a stress induced LncRNA which regulates apoptosis and functionally contributes in psoriasis susceptibility^32^,^33^. Studies also support that HIF as a key LncRNA regulator in cancer development. A study conducted by Choudhry *et al.* show that HIF-2α dependent transcriptional activation of NEAT1 contributes to breast cancer tumorigenesis^34^. Further, HIF-1α-responsive lincRNA-p21 and lncRNA-UCA1 is crucial to hypoxia-enhanced glycolysis^35^ and bladder cancer progression^36^ respectively.

Recent reports on LncRNAs provide evidence for their role in cytokine regulation. LincRNA1992 -THRIL is highly associated with Kawasaki disease and regulates immune responses through TNF-α^37^. TH2-LCR LncRNA induces TH-lineage specific cytokines IL-4, IL-5 and IL-13 and regulates T-helper cell differentiation^38^. Lnc-IL7R is involved in the early immune response through regulation of E-selectin, VCAM-1, IL-8, and IL-6 expression^39^. LncRNA- AK139328 knockdown ameliorates liver ischemia/reperfusion injury by reducing inflammatory cytokines^30^. We in the present study demonstrate that LncRNA-PRINS had specific interaction with RANTES and both were significantly up regulated under hypoxic conditions. Further, we provide evidence that HIF-1α is the important mediator in RANTES production through LncRNA regulation. Together, we present for the first time a link between HIF-1α responsive LncRNA in cytokine regulation. However, exact mechanism has to be elucidated; nevertheless these findings provide novel evidence for HIF-1α regulated PRINS in RANTES regulation.

In conclusion, the study demonstrates RANTES as a crucial mediator in acute kidney injury following IR. RANTES mediates AKI through post-ischemia inflammatory reaction constituting ischemic renal tubular cells, infiltrating leukocytes and pro-inflammatory cytokines. Further, RANTES expression is regulated by NF-κB and PRINS might act as a HIF-1α dependent LncRNA and involve in the process of AKI. Thus, HIF-1α-LncRNA-PRINS-RANTES axis might play a regulatory role in AKI.

## Materials and Methods

### Experimental Animals and Protocol

Male wild-type C57BL/6C (B6) mice were purchased from BioLASCO Taiwan Co, Ltd (Taipei, Taiwan), and RANTES^−/−^ mice were obtained from the Jackson Laboratory (Bar Harbor, ME, USA); their background has been described previously^40^. The mice were maintained under specific pathogen-free housing conditions in China Medical University. Male mice aged 6-8 weeks and weighing 15–20 g were used in the experiments. All animal procedures were approved by Animal Ethics Committee of China Medical University (CMU-101-100-N) and carried out in accordance with the guidelines of Animal Care and Use Guidelines of the China Medical University. The procedure used to induce kidney IRI in the experiments has been described previously^41^. In brief, the mice were anesthetized with ketamine (100 mg/kg intraperitoneally), xylazine (10 mg/kg intraperitoneally), and acepromazine (1 mg/kg intramuscularly), and then subjected to a midline abdominal incision. After general anesthesia, both renal pedicles were clamped for 35 minutes with microaneurysm clamps. After removal of the clamps, the kidneys were observed for 1 minute for restoration of blood flow. Kidneys that did not return to their original color after unclamping were not used for analysis. During the procedure, warm saline was instilled into the peritoneal cavity. Abdominal wounds were closed and examined daily, and animals with signs of wound infection or dehiscence were excluded from the study. All mice were returned to individual ventilated cages with free access to food and water. Sham surgery was performed using the same experimental procedures, except that the renal vessels were not clamped. The mice were sacrificed 1, 3 and 7 days after reperfusion.

### Histology Examination

Histology was performed and all the groups were analyzed for tubular injury.

### Assessment of Renal Function

Serum creatinine was measured using an Astra Auto analyzer (Beckman Instruments, Fullerton, CA).

### Immunohistochemistry (IHC) Staining

Immumohistochemical staining was performed to detect the expression of RANTES, neutrophils (Ly6G), lymphocytes (CD3^+^) and macrophages (F4/80).

### Quantitative real-time PCR

Total RNA was isolated, cDNA was synthesized and qPCR was performed using a RealTime ready assay (TNF-α, IL-1β, MCP-1, GAPDH), with the gene expression analysis done using a Universal ProbeLibrary System on a LightCycler® 480 System (Roche). LncRNA expression (PRINS and DLG2AS1) were analyzed using TaqMan® Non-coding RNA Assays (Life technologies).

### Western blot

Western blot was performed to analyze the expression of RANTES.

### Nuclear Extraction and Electrophoretic Mobility Shift Assay (EMSA)

NF-κB DNA binding activity was measured by EMSA.

### shRNA HIF- 1α knockdown by viral infection

HK-2 were infected with lentivirus expressing shRNA for HIF-1α in the presence of 8 μg/ml protamine sulfate for 24 h, followed by puromycin (2 μg/ml; 48 h) selection. shLacZ which targets the LacZ gene, was used as a control. The knockdown efficiency of HIF-1α was examined using Q-PCR (Biometra T1 thermo cycler).

### LncRNA profiling

Differentially expressed Lnc-RNAs in HK-2 cells and sh-HIF-1α HK-2 cells were identified using Human Disease-Related LncRNA Profiler (CAT# RA920D, System Biosciences).

### Dual-luciferase reporter assay

The HK-2 cells were transfected with 1μg of PRINS vector or control vector for 24 hours and exposed to normoxia/hypoxic conditions. After treatment, the cells were harvested, lysed and assay was carried out as per manufacturer’s protocol (Dual-Luciferase Reporter Assay System, Promega, Madison, WI).

### Statistical analysis

All the data are expressed as mean ± SD unless otherwise specified. Comparisons among multiple groups were performed using 1- or 2-way ANOVA. Statistical differences between 2 groups were calculated using an unpaired Student’s *t* test (2-tailed). *P* values less than 0.05, 0.01, 0.001 were considered significant.

## Additional Information

**How to cite this article**: Yu, T.-M. *et al.* RANTES mediates kidney ischemia reperfusion injury through a possible role of HIF-1a and LncRNA PRINS. *Sci. Rep.* **6**, 18424; doi: 10.1038/srep18424 (2016).

## Supplementary Material

Supplementary Information
