# Supplementary material for: RANTES mediates kidney ischemia reperfusion injury through a possible role of HIF-1α and LncRNA PRINS
Source: Sci Rep. 2016 Jan 4;6:18424. doi: 10.1038/srep18424 (PMC4698731; doi:10.1038/srep18424)
Supplement: Supplementary Information [file srep18424-s1.doc]

**RANTES mediates kidney ischemia reperfusion injury through a possible role of HIF-1α and LncRNA PRINS**

Tung-Min Yu MD PhD^1,2^, Kalaiselvi Palanisamy PhD^1^, Kuo-Ting Sun MD PhD ^1,3,4^, Yuan-Ji Day MD PhD^5,6^, Kuo-Hsiung Shu MD ^2^, I-Kuan Wang MD PhD ^1,7^, Woei-Cherng Shyu MD^8,9^, Ping Chen MD PhD^10^, Yuh-Lien Chen PhD^11^, Chi-Yuan Li MD ^1,12^*

**Materials and Methods**

**Histology Examination**

The kidneys were fixed in periodate-lysine paraformaldehyde (4% paraformaldehyde) and embedded in paraffin, and then cut into 4-μm thick sections. The sections were subjected to routine staining with hematoxylin and eosin stain (H&E) and viewed under a light microscope. Histological examinations were performed by a renal pathologist in a blinded fashion. The changes in tubular necrosis in the outer stripe of the outer medulla were calculated by counting the percentage of tubules that displayed cell necrosis, loss of brush border, cast formation and tubule dilatation. The severity of necrosis was quantitated by scoring the degree of renal injury based on the following scoring system: 0=none, 1=<10%, 2=11 to 25%, 3=26 to 45%, 4=46 to 75%, and 5=>76%. At least 10 high-power fields (magnification, x200) per section for each sample were examined.

**Assessment of Renal Function**

Serum creatinine was measured using an Astra Auto analyzer (Beckman Instruments, Fullerton, CA) by the Biochemistry Department of the China Medical University (Taichung, Taiwan).

**Immunohistochemistry (IHC) Staining**

The 4-μm thick paraffin sections were stained using an automatic immunostaining device (Ventana XT Medical System, Tucson, AZ) and ultraView detection kit (Ventana Medical Systems Inc., Tucson, AZ). All tissue sections were processed using the following primary antibodies: rabbit anti-mouse RANTES antibody (Epitomics Inc., CA, USA); rat anti-mouse F4/80 antibody (Gene Tex Inc., USA); rat anti-mouse Ly-6G antibody (Abcam, USA); and rabbit anti-human CD3 (ScyTek Laboratories Inc.). A standard immunoalkaline phosphatase method was employed, preceded by pressure-cook antigen retrieval for 5 min in Ventana retrieval buffer (pH 10.0; Ventana Medical Systems, Tucson, AZ, USA). Concentration-matched rat IgG was used as an isotype-negative control. Briefly, formalin-fixed and paraffin-embedded tissue array specimens were washed in Tris-buffered saline containing 0.1% Tween-20, rehydrated through serial dilutions of alcohol, and washed in phosphate buffered saline (pH 7.2). The buffer was used for all subsequent washes, according to the manufacturer’s recommended protocol. 3, 3’- diaminobenzidinetetrahydrochloride (Sigma-Aldrich, St. Louis, MO) was used as a chromogen in immunostaining procedures. The sections were counterstained with hematoxylin. Ten fields from the outer medulla were evaluated, scored and averaged by using Image-Pro Plus version 5.0.

**Extraction of RNA and cDNA synthesis**

Total RNA was isolated from mouse kidney tissue using a High Pure RNA Isolation Kit (Roche Applied Sciences, Mannheim, Germany). cDNA was synthesized from 2 ug of RNA using a Transcriptor Frist Strand cDNA Synthesis Kit (Roche). A denature step of 10 minutes at 65°C was generally performed, except in one experiment. Oligo-dT primers were used for the synthesis, which was performed for 60 minutes at 50°C in 20 ul of reaction volume. The cDNA was used immediately for quantitative real-time polymerase chain reactions (qPCR) or stored at -20°C until it was used.

**Quantitative real-time PCR**

Q-PCR was performed using RealTime ready assay (TNF-α, IL-1β, MCP-1, GAPDH), with the gene expression analysis done using a Universal ProbeLibrary System on a LightCycler® 480 System (Roche), according to the manufacturer’s instructions. The reaction conditions were: 95°C for 10 minutes, 45 cycles of 95°C for 10 seconds, 60°C for 30 seconds, and 72°C for 1 second (with single acquisition), followed by 40°C for 30 seconds final cooling. Real-time PCR experiments for each gene were performed as 3 separate experiments. The expression of each gene was normalized against the mRNA expression of the housekeeping gene GAPDH, using the Advanced Relative Quantification of the LightCycler® 480 Software. Data were analyzed using the LightCycler®480 Software version 1.5.0.

The primers used were as follows:

TNF-α (forward, 5’-ACTCCAGGCGGTGCCTATGT-3’; reverse, 5’-TCCAGCTGCTCCTCCACTTG-3’);

MCP-1 (forward, 5’-GAGCATCCACGTGTTGGCT-3’; reverse, 5’-TGGTGAATGAGTAGCAGCAGGT-3’);

IL-1β (forward, 5′-GCACACCCACCCTGCAG-3′; reverse, 5′-AACCGCTTTTCCATCTTCTTCTT-3′)

CCL5 (Forward, 5’- TGCCCACATCAAGGAGTATTT- 3’; reverse, 5’- TTTCGGGTGACAAAGACG-3’

**LncRNA-PRINS and DLG2AS1 expression**

For analysis of LncRNA expression, total RNA was extracted, reverse transcribed and cDNA was analyzed using TaqMan® Non-coding RNA Assays (Life technologies) according to manufacturer’s instructions. The samples were normalized with RPLP0.

***In vitro* hypoxic treatment**

Kidney tubular epithelial cells (NRK-52E, HK-2) were treated in a Biospherix C-Chamber (Biospherix) inside a standard culture chamber by means of exhausting and filling with 95% N_2_ and 5% CO_2_ to produce oxygen concentrations of 0.1 to 1% for 4 h at 37°C to achieve non-interrupted hypoxic conditions.

**Western blot**

RANTES protein expression was confirmed by Western blot analysis. For hypoxia-induced RANTES expression, the cells were exposed to hypoxic stress for 6 and 16 hours. For inhibitor study, the cells were pre-treated with NF-kB inhibitor (BAY 11-7085; Sigma) (5uM) followed by hypoxic stress for 6 and 16 hours. After complete treatment schedule, the proteins were extracted using lysis buffer. The lysates were centrifuged, and aliquots containing 50 μg of protein were separated on SDS PAGE (10%) and blotted on PVDF membranes. Immuno detection was carried out using primary antibodies directed against RANTES (Santa Cruz, sc-1410). Blots were blocked with 5% skim milk-TBS-Tween-20 for 1 h at room temperature and probed with primary antibodies (1:500) against RANTES (Santa Cruz, sc-1410) and β-actin (Sigma-Aldrich) overnight at 4°C. Followed by which blots were washed with PBS-Tween 20 and incubated with horseradish peroxidase-conjugated secondary antibodies (1:1000) for 1 hr at room temperature. The immunoreactive band was detected by enhanced chemiluminescence.

**Nuclear Extraction and Electrophoretic Mobility Shift Assay (EMSA)**

NF-κB DNA binding activity was measured by EMSA. Briefly, nuclear proteins were extracted using a Panomics Nuclear Extraction Kit (Panomics Inc., Fremont, CA) following the manufacturer’s instructions. Protein concentration was measured with Bio-Rad protein assay reagent (Bio-Rad Laboratories, Hercules, CA). The double-strand NF-κB consensus oligonucleotide (Panomics) was 3′-biotinylated with a commercial biotin 3′-end labeling kit (Thermo Scientific, Rockfield, IL). Standard electrophoretic mobility shift assay (EMSA) reactions were conducted with a LightShift™ chemiluminescent EMSA kit (Thermo) with 10 μg of cell nuclear extract and 30 fmol of biotin end-labeled DNA in an 18-μl volume binding reaction per sample, with 5 μl of EMSA buffer (mM: 400 KCl, 80 HEPES, and 0.1 EDTA, with 80% glycerol, pH 8.0), 1 μl of poly(dI-dC) (0.01 U/μl), and 1 μl of sonicated salmon sperm DNA (500 ng/μl). EMSA reactions were carried out at room temperature for 30 min, then terminated by adding 5 μl of 5× nucleic acid sample loading buffer (0.2% bromophenol blue, 0.2% xylene cyanol FF, 25% glycerol, 50 mMTris·HCl, pH 8.0, 5 mM EDTA), and then separated on 6% polyacrylamide gel. The gel was pre-run at 100 V for 30 min and subsequently run at 100 V for 1 h after sample loading in 0.5× TBE buffer (89 mMTris-acetate, 89 mM boric acid, 2 mM EDTA). The reaction products were then transferred to an Immobilon-NC membrane (Millipore Corp, Bedford, MA) at 100 V for 1 h and fixed by UV cross-linking. The biotin-labeled reaction products were then visualized by incubation with streptavidin-horseradish peroxidase (HRP) conjugate and subsequent incubation with SuperSignal West Pico chemiluminescence reagents (Thermo Scientific).

**shRNA knockdown by viral infection**

Human embryonic kidney 293T (HEK293T) cells were cultured as described elsewhere^42^. HK-2 were infected with lentivirus expressing shRNA for HIF-1α in the presence of 8 μg/ml protamine sulfate for 24 h, followed by puromycin (2 μg/ml; 48 h) selection. shLacZ which targets the LacZ gene, was used as a control. The knockdown efficiency of HIF-1α was examined using Q-PCR (Biometra T1 thermo cycler).

**Dual-luciferase reporter assay**

Wild type HK-2 cells were seeded into 6 well plates 2x10^5^ and allowed for overnight attachment. The cells were transfected with 1μg of PRINS vector or control vector for 24 hrs. Followed by which, media was refreshed and incubated under normoxia/hypoxic conditions. After treatment, the cells were harvested, lysed and assay was carried out as per manufacturer’s protocol (Dual-Luciferase Reporter Assay System, Promega, Madison, WI). The results were expressed in luciferase activity normalized with Renilla.

**LncRNA profiling**

Differentially expressed Lnc-RNAs in HK-2 cells and sh-HIF-1α HK-2 cells were identified using Human Disease-Related LncRNA Profiler (CAT# RA920D, System Biosciences). Followed by treatment schedule, RNA was extracted with Trizol Reagent (Invitrogen) and quantified using NanoDrop 2000 spectrophotometer (Thermo Scientific Waltham, MA,). Total RNA was further converted into cDNA and q-PCR was performed to identify the Lnc-RNA expressions and their relative expressions were calculated with the internal control.
